# Neuro-pediatric emergencies: clinical profile and outcomes

**DOI:** 10.25122/jml-2023-0476

**Published:** 2024-04

**Authors:** Imad Khojah, Osama Muthaffar, Hassan Alalawi, Anas Alyazidi, Maha Alghamdi, Ohud Alharbi, Latifa Almuharib, Mayar Alhuqaili

**Affiliations:** 1 Department of Emergency Medicine, King Abdulaziz University, Jeddah, Saudi Arabia; 2 Department of Pediatrics, Division of Pediatrics Neurology, King Abdulaziz University, Jeddah, Saudi Arabia; 3 Faculty of Medicine, King Abdulaziz University, Jeddah, Saudi Arabia; 4 Department of Internal Medicine, King Faisal Specialist Hospital and Research Center, Jeddah, Saudi Arabia; 5 Faculty of Medicine, Qassim University, Al-Mulida, Saudi Arabia; 6 National Guard Riyadh, King Abdullah Specialist Children Hospital, Riyadh, Saudi Arabia

**Keywords:** pediatrics, emergency medicine, chief complaint, neurology, Saudi Arabia

## Abstract

Pediatric neurological emergencies are a significant concern, often leading to high rates of admission to pediatric intensive care units and increased mortality rates. In Saudi Arabia, the emergency department (ED) is the main entry point for most patients in the healthcare system. This study aimed to provide a comprehensive overview of pediatric neurology visits to the ED, analyzing patient demographics, clinical presentations, and outcomes. The retrospective study was conducted at a large tertiary care center and examined 960 pediatric patients with neurological emergencies out of 24,088 pediatric ED visits. The study population consisted mainly of male participants (56.5%) and 43.5% female participants, with a mean age of 5.29 ± 4.19 years. School-age children (6–12 years) represented the largest population group (29.1%), and over a third of patients were triaged as 'resuscitation' (*n* = 332, 34.6%). Seizures (*n* = 317, 33.0%) and postictal states (*n* = 187, 19.5%) were the most common reasons for seeking emergency care, accounting for over half of all cases. There were statistically significant differences in provisional diagnosis and chief complaints across different age groups (*P* >0.001 and *P* <0.001, respectively). The most common outcome was discharge (*n* = 558; 58.1%), and the mean length of stay was 10.56 ± 20.33 hours. Neuro-emergencies in pediatrics are a concern and a leading cause of mortality, morbidities, and increased hospital visits. The observed variations in presentation and outcomes across age groups further emphasize the importance of tailored approaches.

## INTRODUCTION

Emergency departments (ED) are the primary point of contact for hospital care in many situations, including Saudi Arabia, where these serve as the main entry point to the healthcare system for most patients [1,2]. Moreover, despite the severity or complexity of the primary medical complaint, pediatric patients continue to receive care from the ED frequently. This often leads to overcrowding and prolonged waiting hours, which can impact the quality and quantity of care [3]. Among these pediatric ED visits, neurological emergencies represent a critical subset, encompassing severe and life-threatening conditions like epileptic conditions, stroke, central nervous system infections, and neurosurgical emergencies that require special attention and intensive care. Although these cases represent a small percentage of overall pediatric ED visits (2-15%) [4–6], they account for nearly three times the frequency of pediatric intensive care unit (PICU) admissions, with a mortality rate that can be up to 50% [7]. A progressive increase in ED visits is observable, especially among communities with chronic neurological diseases and among growing populations with greater risk factors and comorbidities [8]. Children with neurological emergencies present with a variety of symptoms when they arrive in the ED [9]. In coastal regions such as Jeddah, drowning can also be a significant contributor to both severe neurologic morbidity and accidental death [10]. Globally, demographic and clinical data regarding pediatric neuro-emergency visits vary among countries [4,11]. Specific data on pediatric neuro-emergencies in our local literature is extremely limited. Only a few reports assessed and explored the characteristics and clinical profile of certain disorders, such as status epilepticus, or were limited to a specific year period [12–15]. This study aimed to address this gap by providing a comprehensive overview of pediatric neurology visits to the ED in the local context. This research aims to inform strategies for maximizing care quality and improving healthcare staff training by analyzing patient demographics, clinical profiles, and outcomes. This comprehensive understanding is crucial for optimizing the management of pediatric neurological emergencies in the region.

## MATERIAL AND METHODS

### Study design and setting

**Figure 1:**
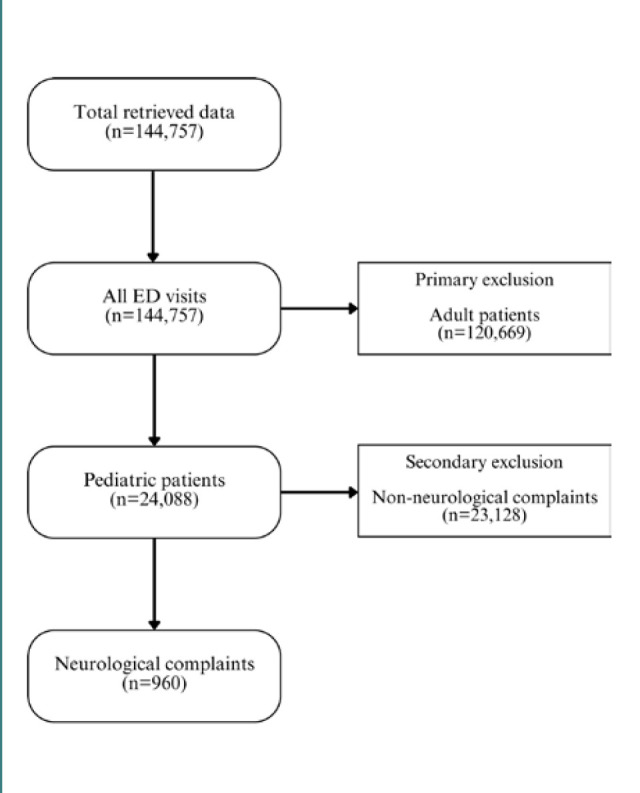
Flow chart of the inclusion criteria

This descriptive, retrospective cohort study adhered to the Strengthening the Reporting of Observational Studies in Epidemiology (STROBE) checklist [16]. The study was conducted in a publicly operated tertiary care center that is funded and owned by the community and serves the entire community with a bed capacity of 750 beds and up to 900 beds in an emergency setting. The center receives an estimated 60,000 visits annually. The data for the emergency department record from 2019–2021 were retrieved directly from the hospital record to limit data entry errors. A total of 144,757 visits were retrieved; however, only pediatric patients were included in this study. Pediatric patients were defined as patients aged 14 years old or younger, all of whom were included. No other restrictions were applied at that stage. After including 24,088 pediatric patients, they were screened based on preliminary diagnosis (Figure 1). Patients with a neuro-related provisional diagnosis were included after identifying the clinical characteristics and assessing the severity of clinical presentation across multiple variables. The study aimed to correlate triage level with presentation severity and admission type. Finally, the large sample size contributed to the reliability of the study because every visit was included.

### Statistical analysis

The following variables were extracted: gender, age, nationality, provisional diagnosis, chief complaint, outcome, triage level, visit time, length of stay (LOS), date of admission, number of X-rays, magnetic resonance imaging (MRI), computerized tomography (CT), lab workups, and number of medications. Statistical analysis was performed using the Statistical Package for the Social Sciences (SPSS) version 26 (IBM Corp., Armonk, NY, USA) and SmartPLS 3 to test the relationship between the variables. Variables were distributed into qualitative and quantitative categories. Qualitative variables were described in frequency tables with percentages, and quantitative data were described with the mean and standard deviation (SD). The chi-square test was used to compare categorical variables. One-way analysis of variance (ANOVA) and multinomial logistic regression were used to predict the difference between demographic and clinical characteristics. All data utilized graphical presentation in the form of line charts and illustrated graphs. *P* values <0.05 were considered statistically significant.

## RESULTS

### Demographic and clinical characteristics

The study included 960 patients with a provisional neurological diagnosis. Table 1 describes patients' baseline characteristics. There were 542 (56.5%) male and 418 (43.5%) female patients. The average age of the patients was 5.29 ± 4.19 years, with the school-age group (6–12 years) being the most prevalent, comprising 279 (29.1%) of the total patients. Most patients were triaged as ‘priority 1: resuscitation’ (*n* = 332; 34.6%), followed by ‘priority 3: urgent’ (*n* = 331, 34.5%). Table 2 presents a comparison of various characteristics stratified by gender, while Table 3 provides a comparison based on different age groups.

**Table 1:** Baseline characteristics of study participants

Characteristic	
Age, in years (mean ± SD)	5.29 ± 4.19
**Age groups *n* (%)**	
Neonate (birth – 1 month)	25 (2.6)
Infancy (1 month – 1 year)	163 (17)
Toddler (1–3 years)	196 (20.4)
Preschool (3–6 years)	200 (20.8)
School-age (6–12 years)	279 (29.1)
Adolescent (12–14 years)	97 (10.1)
**Gender *n* (%)**	
Male	542 (56.5)
Female	418 (43.5)
**Nationality *n* (%)**	
Saudi	447 (46.6)
Non-Saudi	513 (53.4)
**Triage *n* (%)**	
Priority 1 - Resuscitation	332 (34.6)
Priority 2 - Emergent	280 (29.2)
Priority 3 - Urgent	331 (34.5)
Priority 4 - Less urgent	11 (1.1)
Priority 5 - Non-urgent	6 (0.6)
**Outcome *n* (%)**	
Discharged	558 (58.1)
Admitted	394 (41.0)
AMA (against medical advice)	4 (0.4)
LBT (left before treatment)	3 (0.3)
Deceased (hospital death)	1 (0.1)
**Provisional diagnosis *n* (%)**	
Seizure/Epilepsy	529 (55.1)
Headache/Loss of consciousness	23 (2.4)
Inflammation	59 (6.1)
Cranial nerve palsy	59 (6.1)
Neuromuscular	14 (1.5)
Neoplasm	55 (5.7)
Vascular	92 (9.6)
Injury	128 (13.3)
Abscess	1 (0.1)
**Most common chief complaints *n* (%)**	
Seizures	317 (33.0)
Post-ictal	187 (19.5)
Fever	84 (8.8)
Head injury	75 (7.8)
Shortness of breath	35 (3.6)
Vomiting	35 (3.6)
Trauma	29 (3.0)
Headache	26 (2.7)
Weakness	23 (2.4)
Swelling	20 (2.1)
**Length of stay *n* (%)**	
Less than 24 hours	896 (93.3%)
24-48 hours	39 (4.1%)
More than 48 hours	25 (2.6%)
Length of stay in hours (mean ± SD)	10.56 ± 20.33
**Investigations (mean ± SD)**	
X-ray	0.48 ± 0.94
CT scan	0.50 ± 0.77
MRI	0.05 ± 0.34
Labs	8.53 ± 6.39
Medications	3.19 ± 4.15

**Table 2:** Baseline characteristics and clinical presentations by gender

Characteristic	Overall (*n* = 960)	Male (*n* = 542)	Female (*n* = 418)	*P* value
Age, in years (mean ± SD)	5.29 ± 4.19	5.04 ± 3.90	5.60 ± 4.54	0.040
**Age groups**				**0.000**
Neonate (birth – 1 month)	25 (2.6)	14 (2.6)	11 (2.6)	
Infant (1 month – 1 year)	163 (17)	87 (16.1)	76 (18.2)	
Toddler (1-3 years)	196 (20.4)	122 (22.5)	74 (17.7)	
Preschool (3-6 years)	200 (20.8)	110 (20.3)	90 (21.5)	
School-age (6-12 years)	279 (29.1)	175 (32.3)	104 (24.9)	
Adolescent (12-14 years)	97 (10.1)	34 (6.3)	63 (15.1)	
**Nationality *n* (%)**				**0.683**
Saudi	447 (46.6)	256 (47.2)	191 (45.7)	
Non-Saudi	513 (53.4)	286 (52.8)	227 (54.3)	
**Triage *n* (%)**				**0.649**
Priority 1 - Resuscitation	332 (34.6)	180 (33.2)	152 (36.4)	
Priority 2 - Emergent	280 (29.2)	167 (30.8)	113 (27.0)	
Priority 3 - Urgent	331 (34.5)	187 (34.5)	144 (34.4)	
Priority 4 - Less urgent	11 (1.1)	5 (0.9)	6 (1.4)	
Priority 5 - Non-urgent	6 (0.6)	3 (0.6)	3 (0.7)	
**Outcome *n* (%)**				**0.531**
Discharged	558 (58.1)	314 (57.9)	244 (58.4)	
Admitted	394 (41.0)	222 (41.0)	172 (41.1)	
AMA (against medical advice)	4 (0.4)	2 (0.4)	2 (0.5)	
LBT (left before treatment)	3 (0.3)	3 (0.6)	0 (0.0)	
Deceased (hospital death)	1 (0.1)	1 (0.2)	0 (0.0)	
**Provisional diagnosis *n* (%)**				**0.454**
Seizure/Epilepsy	529 (55.1)	303 (55.9)	226 (54.1)	
Headache/Loss of consciousness	23 (2.4)	17 (3.1)	6 (1.4)	
Inflammation	59 (6.1)	31 (5.7)	28 (6.7)	
Cranial nerve palsy	59 (6.1)	30 (5.5)	29 (6.9)	
Neuromuscular	14 (1.5)	8 (1.5)	6 (1.4)	
Neoplasm	55 (5.7)	26 (4.8)	29 (6.9)	
Vascular	92 (9.6)	51 (9.4)	41 (9.8)	
Injury	128 (13.3)	76 (14.0)	52 (12.4)	
Abscess	1 (0.1)	0 (0.0)	1 (0.2)	
**Most common complaints *n* (%)**				**0.346**
Seizures	317 (33.0)	174 (32.1)	143 (32.1)	
Post-ictal	187 (19.5)	118 (21.8)	69 (16.5)	
Fever	84 (8.8)	54 (10.0)	30 (7.2)	
Head injury	75 (7.8)	46 (8.5)	19 (6.9)	
Shortness of breath	35 (3.6)	19 (3.5)	16 (3.8)	
Vomiting	35 (3.6)	18 (3.3)	17 (4.1)	
Trauma	29 (3.0)	15 (2.8)	15 (3.3)	
Headache	26 (2.7)	14 (2.6)	12 (2.9)	
Weakness	23 (2.4)	10 (1.8)	13 (3.1)	
Swelling	20 (2.1)	9 (1.7)	11 (2.6)	
**Length of stay**				**0.071**
Less than 24 hours	896 (93.3%)	499 (92.1)	397 (95.0)	
24–48 hours	39 (4.1%)	29 (5.4)	10 (2.4)	
More than 48 hours	25 (2.6%)	14 (2.6)	11 (2.6)	
Length of stay in hours	10.56 ± 20.33	10.69 ± 21.93	10.39 ± 18.08	**0.826**
**Investigations (mean ± SD)**				
X-ray	0.48 ± 0.94	0.46 ± 0.98	0.423 ± 0.88	0.589
CT scan	0.50 ± 0.77	0.51 ± 0.79	0.49 ± 0.74	0.744
MRI	0.05 ± 0.34	0.06 ± 0.37	0.03 ± 0.28	0.341
Labs	8.53 ± 6.39	8.33 ± 6.17	8.79 ± 6.67	0.267
Medications	3.19 ± 4.15	3.16 ± 4.15	3.23 ± 4.15	0.817

**Table 3:** Baseline characteristics and clinical presentations by age group

Characteristic	Overall (*n* = 960)	Neonate (birth–1 month)	Infancy (1 month– 1 year)	Toddler (1–3 years)	Preschool (3–6 years)	School-age (6–12 years)	Adolescent (12–14 years)	*P* value
Age, in years (mean ± SD)	5.29 ± 4.19	0.30 ± 0.031	0.54 ± 0.26	1.98 ± 0.61	4.46 ± 0.88	8.71 ± 1.60	13.15 ± 0.66	0.000
**Gender *n* (%)**								**0.000**
Male	542 (56.5)	14 (56.0)	87 (53.4)	122 (62.2)	110 (55.0)	175 (62.7)	34 (35.1)	
Female	418 (43.5)	11 (44.0)	76 (46.6)	74 (37.8)	90 (45.0)	104 (37.3)	63 (64.9)	
Nationality *n* (%)								0.000
Saudi	447 (46.6)	7 (28.0)	93 (57.1)	106 (54.1)	87 (43.5)	120 (43.0)	34 (35.1)	
Non-Saudi	513 (53.4)	18 (72.0)	70 (42.9)	90 (45.9)	113 (56.5)	159 (57.0)	63 (64.9)	
**Triage *n* (%)**								**0.000**
Priority 1 - Resuscitation	332 (34.6)	4 (16.0)	37 (22.7)	66 (33.7)	71 (35.5)	102 (36.6)	52 (53.6)	
Priority 2 - Emergent	280 (29.2)	18 (72.0)	60 (36.8)	50 (25.5)	60 (30.0)	75 (26.9)	17 (17.5)	
Priority 3 - Urgent	331 (34.5)	3 (12.0)	64 (39.3)	76 (38.8)	65 (32.5)	96 (34.4)	27 (27.8)	
Priority 4 - Less urgent	11 (1.1)	0 (0.0)	2 (1.2)	1 (0.5)	4 (2.0)	3 (1.1)	1 (1.0)	
Priority 5 - Non-urgent	6 (0.6)	0 (0.0)	0 (0.0)	3 (1.5)	0 (0.0)	3 (1.1)	0 (0.0)	
**Outcome *n* (%)**								**0.000**
Discharged	558 (58.1)	1 (4.0)	69 (42.3)	112 (57.1)	116 (58.0)	184 (65.9)	76 (78.4)	
Admitted	394 (41.0)	24 (96.0)	93 (57.1)	82 (41.8)	82 (41.0)	92 (33.0)	21 (21.6)	
AMA (against medical advice)	4 (0.4)	0 (0.0)	1 (0.6)	2 (1.0)	0 (0.0)	1 (0.4)	0 (0.0)	
LBT (left before treatment)	3 (0.3)	0 (0.0)	0 (0.0)	0 (0.0)	2 (1.0)	1 (0.4)	0 (0.0)	
Deceased (hospital death)	1 (0.1)	0 (0.0)	0 (0.0)	0 (0.0)	0 (0.0)	1 (0.4)	0 (0.0)	
**Provisional diagnosis *n* (%)**								**0.000**
Seizure/Epilepsy	529 (55.1)	10 (40.0)	81 (49.7)	101 (51.5)	109 (54.5)	160 (57.3)	68 (70.1)	
Headache/Loss of consciousness	23 (2.4)	0 (0.0)	4 (2.5)	4 (2.0)	4 (2.0)	4 (1.4)	7 (7.2)	
Inflammation	59 (6.1)	11 (44.0)	20 (12.3)	9 (4.6)	8 (4.0)	8 (2.9)	3 (3.1)	
Cranial nerve palsy	59 (6.1)	0 (0.0)	1 (0.6)	8 (4.1)	26 (13.0)	18 (6.5)	6 (6.2)	
Neuromuscular	14 (1.5)	0 (0.0)	0 (0.0)	6 (3.1)	2 (1.0)	4 (1.4)	2 (2.1)	
Neoplasm	55 (5.7)	0 (0.0)	0 (0.0)	6 (3.1)	7 (3.5)	35 (12.5)	7 (7.2)	
Vascular	92 (9.6)	4 (16.0)	27 (16.6)	22 (11.2)	20 (10.0)	17 (6.1)	2 (2.1)	
Injury	128 (13.3)	0 (0.0)	30 (23.4)	40 (20.4)	24 (12.0)	32 (11.5)	2 (2.1)	
Abscess	1 (0.1)	0 (0.0)	0 (0.0)	0 (0.0)	0 (0.0)	1 (0.4)	0 (0.0)	
**Most common complaints *n* (%)**								**0.000**
Seizures	317 (33.0)	4 (16.0)	32 (19.6)	61 (31.1)	68 (34.0)	101 (36.2)	51 (52.6)	
Post-ictal	187 (19.5)	3 (12.0)	46 (28.2)	33 (16.8)	39 (19.5)	54 (19.4)	12 (12.4)	
Fever	84 (8.8)	9 (36.0)	20 (12.3)	15 (7.7)	12 (6.0)	22 (7.9)	6 (6.2)	
Head injury	75 (7.8)	0 (0.0)	16 (9.8)	27 (13.8)	13 (6.5)	16 (5.7)	3 (3.1)	
Shortness of breath	35 (3.6)	3 (12.0)	5 (3.1)	8 (4.1)	10 (5.0)	7 (2.5)	2 (2.1)	
Vomiting	35 (3.6)	0 (0.0)	12 (7.4)	6 (3.1)	7 (3.5)	10 (3.6)	0 (0.0)	
Trauma	29 (3.0)	0 (0.0)	7 (4.3)	13 (6.6)	8 (4.0)	1 (0.4)	0 (0.0)	
Headache	26 (2.7)	0 (0.0)	0 (0.0)	1 (0.5)	7 (3.5)	14 (5.0)	4 (4.1)	
Weakness	23 (2.4)	0 (0.0)	1 (0.6)	4 (2.0)	8 (4.0)	7 (2.5)	3 (3.1)	
Swelling	20 (2.1)	2 (8.0)	13 (8.0)	1 (0.5)	2 (1.0)	2 (0.7)	0 (0.0)	
**Length of stay *n* (%)**								**0.776**
Less than 24 hours	896 (93.3%)	24 (96.0)	147 (90.2)	183 (93.4)	187 (93.5)	265 (95.0)	90 (92.8)	
24-48 hours	39 (4.1%)	0 (0.0)	11 (6.7)	9 (4.6)	7 (3.5)	8 (2.9)	4 (4.1)	
More than 48 hours	25 (2.6%)	1 (4.0)	5 (3.1)	4 (2.0)	6 (3.0)	6 (2.2)	3 (3.1)	
Length of stay in hours (mean ± SD)	10.56 ± 20.33	14.97 ± 38.93	13.83 ± 35.84	8.74 ± 11.82	10.61 ± 17.03	9.71 ± 13.78	9.97 ± 10.83	0.185
**Investigations (mean ± SD)**								
X-ray	0.48 ± 0.94	1.28 ± 1.21	0.58 ± 1.21	0.42 ± 0.79	0.46 ± 1.04	0.36 ± 0.78	0.27 ± 0.65	0.000
CT scan	0.50 ± 0.77	0.52 ± 0.87	0.54 ± 0.68	0.60 ± 0.84	0.54 ± 0.79	0.44 ± 0.77	0.33 ± 0.67	0.000
MRI	0.05 ± 0.34	0.04 ± 0.20	0.04 ± 0.19	0.05 ± 0.35	0.04 ± 0.33	0.07 ± 0.37	0.04 ± 0.41	0.954
Labs	8.53 ± 6.39	16.36 ± 7.24	9.49 ± 6.83	7.69 ± 6.06	8.31 ± 5.72	8.36 ± 6.70	7.57 ± 4.95	0.000
Medications	3.19 ± 4.15	6.48 ± 6.27	2.81 ± 3.57	2.79 ± 3.25	3.29 ± 4.88	3.07 ± 3.90	3.92 ± 4.64	0.000

### Patients’ complaints

**Figure 2:**
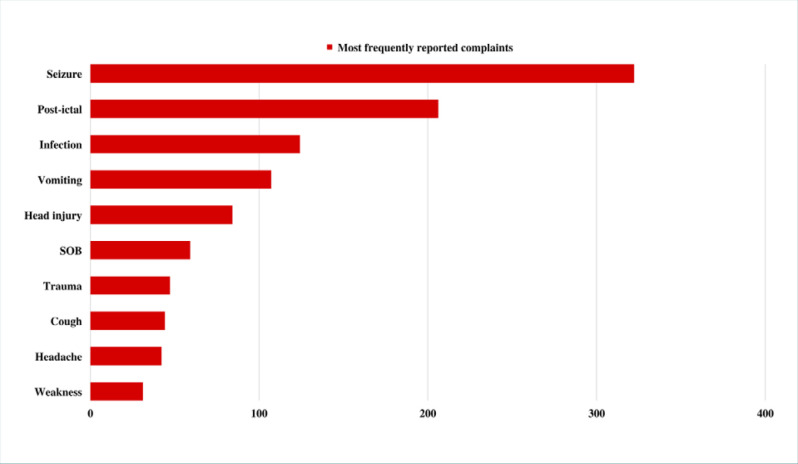
Most commonly reported neurological complaints in pediatric patients

The most frequent chief complaint was seizures (*n* = 317, 33.0%) (Figure 2), followed by post-ictal state (*n* = 187, 19.5%). Most patients received a provisional diagnosis of seizures or epilepsy (*n* = 529, 55.1%). No statistically significant differences in provisional diagnosis or chief complaints were observed between genders (*P* = 0.454 and *P* = 0.346, respectively; Table 2). In contrast, there were statistically significant differences in provisional diagnosis and chief complaints when comparing different age groups (*P* >0.001 and *P* <0.001, respectively; Table 3). Table 4 presents a multinomial logistic regression comparing age groups to a specific provisional diagnosis.

**Table 4:** Multinomial logistic regression analysis of clinical presentations across different age groups

				95% Confidence interval				95% Confidence interval	
	Provisional diagnosis	*P* value	Crude Odds ratio	Lower value	Upper value	*P* value	Adjusted odds ratio	Lower value	Upper value
Neonate	Seizure/Epilepsy	0.182	0.534	0.238	1.202	0.464	1.426	0.551	3.686
	Headache/Loss of consciousness^*^	0.896				0.998			
	Inflammation	0.000	14.519	6.259	33.680	0.000	7.115	2.783	18.187
	Cranial nerve palsy^*^	0.382				0.997			
	Neuromuscular^*^	1.000				0.999			
	Neoplasm^*^	0.416				0.997			
	Vascular	0.447	1.833	0.615	5.461	0.628	0.752	0.238	2.380
	Injury^*^	0.091				0.996			
	Abscess^*^	1.000				1.000			
Infant	Seizure/Epilepsy	0.150	0.770	0.549	1.078	0.284	1.242	0.835	1.847
	Headache/Loss of consciousness	1.000	1.030	0.346	3.069	0.998	1.001	0.316	3.168
	Inflammation	0.001	2.718	1.541	4.797	0.089	1.712	0.921	3.185
	Cranial nerve palsy	0.002	0.079	0.011	0.572	0.006	0.062	0.008	0.459
	Neuromuscular*	0.178				0.999			
	Neoplasm*	0.001				0.997			
	Vascular	0.001	2.236	1.377	3.630	0.049	1.678	1.002	2.810
	Injury	0.050	1.609	1.027	2.521	0.021	1.808	1.094	2.988
	Abscess*	1.000				1.000			
Toddler	Seizure/Epilepsy	0.295	0.835	0.609	1.143	0.152	0.755	0.514	1.109
	Headache/Loss of consciousness	0.918	0.817	0.275	2.429	0.697	0.802	0.263	2.441
	Inflammation	0.396	0.687	0.332	1.423	0.289	0.660	0.306	1.423
	Cranial nerve palsy	0.237	0.595	0.278	1.275	0.191	0.596	0.274	1.294
	Neuromuscular	0.078	2.984	1.023	8.703	0.041	3.122	1.048	9.295
	Neoplasm	0.103	0.461	0.194	1.092	0.118	0.494	0.204	1.196
	Vascular	0.460	1.254	0.755	2.081	0.375	1.275	0.745	2.183
	Injury	0.002	1.970	1.304	2.975	0.001	2.171	1.370	3.441
	Abscess*	1.000				1.000			
Preschool	Seizure/Epilepsy	0.910	0.970	0.709	1.326	0.849	0.964	0.659	1.409
	Headache/Loss of consciousness	0.880	0.796	0.268	2.366	0.581	0.723	0.229	2.284
	Inflammation	0.210	0.579	0.270	1.241	0.128	0.537	0.241	1.197
	Cranial nerve palsy	0.000	3.292	1.918	4.886	0.000	3.479	1.990	6.082
	Neuromuscular	0.782	0.630	0.140	2.836	0.538	0.622	0.137	2.825
	Neoplasm	0.176	0.538	0.240	1.208	0.102	0.500	0.218	1.147
	Vascular	0.928	1.062	0.630	1.789	0.764	1.087	0.630	1.874
	Injury	0.612	0.860	0.535	1.382	0.547	0.854	0.512	1.426
	Abscess*	1.000				1.000			
School-age	Seizure/Epilepsy	0.410	1.137	0.858	1.506	0.850	0.967	0.686	1.365
	Headache/Loss of consciousness	0.310	0.507	0.171	1.503	0.150	0.442	0.145	1.343
	Inflammation	0.010	0.365	0.171	0.779	0.068	0.477	0.216	1.058
	Cranial nerve palsy	0.917	1.077	0.607	1.908	0.723	1.113	0.616	2.011
	Neuromuscular	1.000	0.976	0.304	3.138	0.823	1.144	0.351	3.732
	Neoplasm	0.000	4.741	2.685	8.372	0.000	7.785	4.183	14.488
	Vascular	0.026	0.524	0.304	0.905	0.078	0.599	0.339	1.059
	Injury	0.326	0.789	0.515	1.210	0.102	0.680	0.428	1.080
	Abscess^*^	0.645				1..000			
Adolescent	Seizure/Epilepsy	0.002	2.045	1.298	3.222	0.306	1.347	0.761	2.386
	Headache/Loss of consciousness	0.003	4.117	1.650	10.274	0.000	6.803	2.449	18.896
	Inflammation	0.272	0.460	0.141	1.498	0.924	0.939	0.261	3.382
	Cranial nerve palsy	1.000	1.008	0.421	2.409	0.717	1.188	0.468	3.014
	Neuromuscular	0.939	1.493	0.329	6.771	0.254	2.537	0.512	12.565
	Neoplasm	0.664	1.321	0.580	3.005	0.060	2.398	0.963	5.966
	Vascular	0.013	0.181	0.044	0.746	0.085	0.282	0.067	1.193
	Injury	0.001	0.123	0.030	0.506	0.003	0.113	0.027	0.478
	Abscess	1.000				1.000			

### Patients’ outcomes

**Figure 3:**
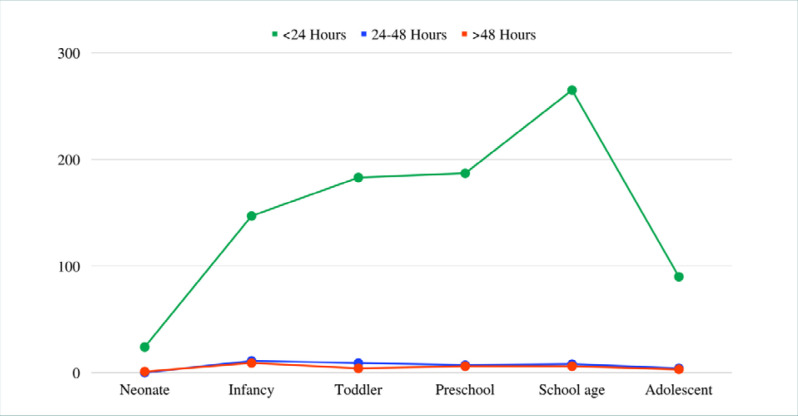
Average length of hospital stay by pediatric age group

The most common outcome was discharge (*n* = 558, 58.1%), followed by admission (*n* = 394, 41.0%). The mean length of stay was 10.56 ± 20.33 hours (Figure 3), and most patients stayed for less than 24 hours (*n* = 896, 93.3%). No significant differences were found between gender and outcomes (*P* = 0.531) or length of stay (*P* = 0.826). Most male and female patients stayed less than 24 hours. However, outcomes varied significantly across age groups (*P* <0.001), while length of stay did not (*P* = 0.776, Table 3).

## DISCUSSION

The primary purpose of this study was to explore the current clinical and demographic characteristics of pediatric neuro-emergencies using hospital data from a tertiary care center. Nationwide, this study serves as the first of its kind to comprehensively review such characteristics. Our study included many age groups, from neonates to adolescents, with 960 patients, including 542 male and 418 female participants. The mean age of patients presenting to the ED in our study was 5.3 years, indicating that the majority of patients present at school age (*n* = 279). In comparison, the male group was larger than the female group. Moreover, seizure was the leading complaint, similar to the literature findings [9]. The majority of patients were triaged as ‘priority 1: resuscitation’ (34.6%). This is likely due to the study setting in a public tertiary care center, which receives many critically ill patients without major prerequisites. Our analysis of patient demographics revealed a significant impact of age on both treatment plans and provisional diagnosis (*P* = 0.000). This finding aligns with existing literature, emphasizing the unique characteristics and needs of different pediatric age groups [17]. Consistent with Kim *et al*. [18], our study found more visits from school-aged children compared to neonates. The admission rate was high in all age groups, particularly newborns requiring supervision and stabilization. This serves as important information, as some studies have reported inequalities in pediatric hospital admissions [19]. Moreover, the number of cases who left against medical advice was low across all age groups, potentially reflecting increased awareness among patients and families regarding the importance of medical care. Also, the most common provisional diagnosis across all groups was seizure, followed by injury and vascular injury. Seizures were more common between ages 3 and 12 and were the second most common in newborns, after inflammation. This supports other studies that concluded that seizures and headaches are the most frequent reasons for admitting pediatric patients presenting to ED with neuro-related complaints [4]. In Saudi Arabia, the prevalence of seizures increased over time, aligning with global trends and surpassing other neurological complaints. This increase is likely due to several risk factors present within the Saudi community, including a higher prevalence of genetic epilepsies due to high rates of consanguineous marriages [20–22]. This can explain the leading trend of seizure in our cohort in comparison to other neurological complaints. However, it is expected for seizures to be prevalent among ED neuro-related complaints, as they can affect 4–10% of the general pediatric population [23,24]. We observed a very low mortality rate of 0.1%, likely attributable to the nature of our non-trauma center where the study was conducted. Nevertheless, even in this setting, life-threatening diagnoses like neuromuscular disorders and neoplasms were observed, underscoring the importance and concern surrounding pediatric neurological emergencies. Cranial nerve palsy was more common in preschoolers than in neonates and infants, while headaches were rare in this age group. Other studies reported a mean age of cranial nerve injuries of 9 ± 6 years [25]. Furthermore, head injuries and neuromuscular injuries were more common in young children, according to preliminary diagnosis, and even between the ages of 1 month and 6 years. Of particular interest is that neoplasia was reasonably rare at young ages but common at school age. As seizures are common as a preliminary diagnosis, this was particularly worrying. Seizures and postictal states were the most common complaints across all age groups but less frequent in school-aged children. Fever was more prevalent in newborns and infants than in older children and adolescents. Infants also experienced head injuries more frequently, although previous studies suggest these injuries are typically benign [26]. As for the frequency of hospital stays, 896 of 960 visits were under 24 hours.

The retrospective study design could impact the results as the outcomes were assessed in a single center. We addressed this limitation by including a large and diverse sample, ensuring representative demographics. Additionally, electronic medical records facilitated access to high-quality data, enhancing the reliability of our findings. Other potential limitations include sample bias and the variability in participant demographics and numbers. Additionally, medication details and imaging protocols were unavailable for review. However, the large sample size and representative demographics help minimize the impact of these limitations on the overall conclusions of the study.

## CONCLUSION

Pediatric neuro-emergencies continue to be a significant concern, contributing to mortality, morbidity, and increased hospital visits and lengths of stay. Variations were observed across different age groups, with consistent patterns emerging within each group, aligning with existing literature. Seizures remain a leading cause of admission compared to other neuro-related complaints. Specific groups demonstrated certain patterns, such as the prevalence of cranial nerve palsy among preschool patients. Expanding the current findings to analyze the characteristics and flow patterns would greatly benefit practitioners and researchers..

## Personal thanks

The authors would like to thank the dedicated healthcare staff for documenting patient information, which was instrumental in completing this study.
